# Clinicopathologic, molecular, and treatment features of metastatic and distantly recurrent extramammary Paget disease: Mayo clinic experience

**DOI:** 10.1093/oncolo/oyag220

**Published:** 2026-06-02

**Authors:** Riham Suleiman, Ray Guo, Mary E Lohman, Stephan D Thome, Lance C Pagliaro, Svetomir N Markovic, Thorvardur R Halfdanarson, Harry E Fuentes Bayne

**Affiliations:** Department of Oncology, Mayo Clinic, Rochester, MN 55905, United States; Department of Laboratory Medicine and Pathology, Mayo Clinic, Jacksonville, FL 32224, United States; Department of Dermatology, Division of Dermatologic Surgery, Mayo Clinic, Rochester, MN 55905, United States; Department of Oncology, Mayo Clinic, Rochester, MN 55905, United States; Department of Oncology, Mayo Clinic, Rochester, MN 55905, United States; Department of Oncology, Mayo Clinic, Rochester, MN 55905, United States; Department of Oncology, Mayo Clinic, Rochester, MN 55905, United States; Department of Oncology, Mayo Clinic, Rochester, MN 55905, United States

**Keywords:** extramammary Paget disease, ERBB2, androgen receptor, tumor biomarkers, next-generation sequencing

## Abstract

**Background:**

Extramammary Paget disease (EMPD) is a rare cutaneous adenocarcinoma. While localized disease has an excellent prognosis, metastatic EMPD is aggressive with no standardized systemic therapy. This study characterizes the clinicopathologic and molecular landscape of metastatic/recurrent EMPD to identify therapeutic vulnerabilities.

**Patients and methods:**

We conducted a retrospective cohort study of 26 patients with metastatic or distantly recurrent EMPD treated at Mayo Clinic (2000-2025). Descriptive statistics were used to summarize baseline characteristics. Survival outcomes were estimated using Kaplan–Meier methods, with group comparisons performed using log-rank testing. Continuous variables were compared using nonparametric tests, and categorical variables were analyzed using appropriate comparative tests. Pathway-level genomic correlations with TCGA reference cohorts were assessed using Spearman rank correlation. Treatment responses were retrospectively evaluated from radiologic reports.

**Results:**

The cohort was predominantly male (73.1%) and White (92.3%), with a median age of 68 (IQR, 53-79). Lymph nodes were the most common metastatic site (61.5%). The median overall survival (OS) was 20.0 months (95% CI, 0-40.4). IHC showed universal CK7 and GATA3 positivity, with 80% androgen receptor (AR) expression. HER2 was positive (3+) or amplified in approximately one-third of cases. NGS revealed frequent mutations in TP53 (68.8%), ERBB2 (50%), and CDKN2A/B (50%). A “triple-hit” deletion at the 9p21 locus (CDKN2A/B/MTAP) was identified in 25% of cases. Genomic profiling showed strong similarity to HER2+ breast and urothelial carcinomas. HER2-directed therapy was associated with longer overall survival (median OS 73.0 months [95% CI, 0-177.9] vs 19.0 months [95% CI, 0-41.4]; HR 0.50 [95% CI, 0.0-177.9], *P* = .3), although this difference did not reach statistical significance.

**Conclusion:**

Metastatic EMPD exhibits distinct clinicopathologic and molecular features, including high AR and HER2 expression, TP53 and CDKN2A/B alterations, and similarity to systemic HER2+ malignancies. The observed activity of HER2-directed therapies in this cohort suggests the potential value of further investigating biomarker-guided treatment strategies in metastatic EMPD. NGS-guided profiling may inform precision treatment strategies, including AR-targeted therapy and emerging MTAP-directed approaches.

Implications for Practice This study underscores the necessity of comprehensive molecular profiling in metastatic EMPD. The high prevalence of HER2 alterations and androgen receptor expression identifies actionable targets that can significantly extend survival beyond traditional chemotherapy. Furthermore, the identification of the 9p21 (CDKN2A/B/MTAP) deletion introduces a novel therapeutic window for CDK4/6 and PRMT5 inhibitors. Clinicians should utilize NGS-guided “basket-trial” strategies, as the genomic similarity to breast and urothelial carcinomas provides a validated roadmap for precision treatment in this rare, aggressive malignancy.

## Introduction

First described by Crocker in 1889,^1^ extramammary Paget disease (EMPD) is a rare cutaneous adenocarcinoma that arises in apocrine gland–bearing skin, most commonly involving the vulva, perineum, scrotum, penis, and perianal skin.^2–4^ The disease typically affects postmenopausal Caucasian women,^5^^,^^6^ though men, particularly in the genital region, are also affected.^7^ Clinically, EMPD manifests as a slowly enlarging erythematous plaque with scaling, erosion, or ulceration.^8^ Because these features resemble benign inflammatory dermatoses, diagnosis is frequently delayed,^6^ contributing to both underrecognition and an uncertain true incidence.^9^

EMPD is classified as either primary or secondary. Primary EMPD represents an intraepidermal adenocarcinoma arising within the skin.^10^ Although its histogenesis remains controversial, proposed cells of origin include intraepidermal pluripotent stem cells,^11^ apocrine or eccrine glandular cells,^11^ and Toker cells.^12^ Reports of multifocal and bilateral cases further support a possible multicentric origin.^13^^,^^14^ In contrast, secondary EMPD results from epidermotropic spread of an underlying visceral malignancy, most commonly of colorectal or urothelial carcinomas.^15^^,^^16^ Differentiating primary from secondary EMPD is essential, as management and prognosis differ significantly, and the presence of coexisting malignancies should be evaluated according to the anatomic site of the primary lesions.^17^ Accordingly, clinical assessment, targeted imaging, immunohistochemistry (IHC), and systematic evaluation for synchronous internal malignancy are integral to initial diagnostic workup.^17^^,^^18^

Most primary EMPD is diagnosed at an in situ stage,^19^ and carries an excellent prognosis when treated surgically.^20^^,^^21^ However, dermal invasion has been reported in approximately 15%-40% of cases in published series,^22^ although the reported frequency varies considerably across studies and may be influenced by differences in case selection, referral patterns, and histopathologic classification. In this context, consistent distinction between microinvasive and deeply invasive disease is important, as microinvasive tumors may demonstrate clinical behavior closer to in situ disease, whereas deeply invasive lesions are more strongly associated with nodal and distant spread.^23^ Once metastatic, EMPD typically follows an aggressive course with poor survival outcomes,^24^ and management remains challenging due to the absence of standardized systemic therapies and limited durable responses to chemotherapy.^25^ Given the limited understanding of molecular drivers and systemic therapy in metastatic EMPD,^26^^,^^27^ this study characterizes metastatic and recurrent EMPD at Mayo Clinic through integrated clinical, histopathologic, and molecular analysis.

## Methods

### Study design and patients’ population

We conducted a retrospective cohort study of patients with metastatic or distantly recurrent EMPD who were diagnosed or treated at Mayo Clinic sites in Rochester, Minnesota; Jacksonville, Florida; Phoenix, Arizona; and the Mayo Clinic Health System between January 1, 2000, and August 31, 2025. Due to the lack of a unique ICD-specific diagnostic code of EMPD, potential cases were initially identified through the Mayo Clinic electronic health records (EHRs) using diagnostic code C44.99 (other specified malignant neoplasm of skin, unspecified). All retrieved records subsequently underwent detailed manual review to confirm eligibility and identify cases of EMPD. Final inclusion required histopathologic confirmation by a board-certified dermatopathologist at Mayo Clinic.

Metastatic or distantly recurrent EMPD was defined as (1) metastatic disease identified at initial presentation or (2) development of distant recurrence following prior definitive local therapy. To ensure consistency across the study period, lymph node involvement beyond expected regional drainage basins, including nodes outside the superficial inguinal, deep inguinal, external iliac, and obturator nodal groups, was retrospectively classified as distant metastatic disease using a standardized anatomic framework. The study was approved by the Mayo Clinic Institutional Review Board.

### Inclusion and exclusion criteria

Eligible patients were 18 years of age or older, had a pathology-confirmed diagnosis of EMPD, and met the definition of metastatic or distantly recurrent disease. Patients were excluded if they had insufficient clinical or pathological documentation, non-EMPD histology, primary mammary Paget disease, or in situ EMPD without evidence of distant spread. A detailed flow diagram of case selection is shown in Figure S1.

### Study variables

EHRs of eligible patients were reviewed, and data were extracted across demographic, clinical, pathological, and treatment domains. Demographic variables included age, sex, race, smoking history, and Eastern Cooperative Oncology Group (ECOG) performance status.

Clinical data included the primary tumor site, clinical presentation, and lesion size. For patients presenting with multiple primary lesions, the total combined tumor size was recorded. Secondary EMPD was assessed using clinical, imaging, endoscopic (when indicated), histopathologic, and immunohistochemical correlation, and diagnosed only in the presence of a concurrent or prior anatomically and histologically compatible internal malignancy. Disease stage at initial diagnosis was retrospectively harmonized across the cohort using a clinical staging framework adapted from Ohara et al.^23^ to improve consistency despite evolving staging systems and imaging practices over the study period.

Histopathological data included IHC staining patterns, molecular testing results, and the depth of invasion. Invasion depth was categorized as in situ (confined to the epidermis), microinvasive (restricted to the papillary dermis, ≤1 mm), or deeply invasive (extending into the reticular dermis or deeper, >1 mm). Human epidermal growth factor receptor 2 (HER2) status was defined using a hierarchical approach: IHC was used as the initial assessment (0-3+ per ASCO/CAP guidelines),^28^ with fluorescence in situ hybridization (FISH) and/or next-generation sequencing (NGS) used for reflex or confirmatory testing in equivocal or selected cases when available.

NGS was performed using clinically validated, targeted tumor sequencing panels applied in routine clinical care at Mayo Clinic. Panel content and platform varied over the study period as sequencing technologies evolved. For comparative genomic analyses, detected somatic alterations were encoded in a binary matrix (1 = any mutation or copy number alteration; 0 = none). Copy number alterations were harmonized across assays using this binary framework (amplification/deletion vs no alteration) to ensure cross-platform comparability. Although all variants were included in the oncoprint, interpretation focused on recurrent, biologically plausible driver alterations, and established cancer-associated genes. Tumor mutational burden (TMB) status was categorized as high (≥10 mutations/Mb) or low (<10 mutations/Mb) based on established thresholds.^29^

Treatment-related variables included systemic therapy regimen, treatment responses, and duration of response defined by treating oncologist.

### Definitions and study endpoints

The primary objectives of this study were to characterize the clinicopathologic and molecular features of metastatic or distantly recurrent EMPD, and to evaluate key clinical endpoints. Primary endpoints included overall survival (OS) and treatment response to systemic therapy. OS was defined as the interval from the diagnosis of metastatic disease to death from any cause or last follow-up. Treatment response was assessed retrospectively through review of radiology reports and clinical documentation, as imaging modalities, scan availability, and reporting practices varied over the study period. Standardized RECIST-based reassessment was not feasible given the retrospective nature of the cohort and the heterogeneity of imaging data across study interval. Responses were categorized as complete response (CR), partial response (PR), stable disease (SD), or progressive disease (PD) based on the interpreting radiologist’s overall assessment. Objective response rate (ORR) was defined as the proportion of patients achieving CR or PR.

Time to distant metastasis was evaluated as a descriptive disease-course endpoint among patients who initially presented with localized disease and subsequently developed distant recurrence. It was calculated from the date of definitive local therapy to the first occurrence of distant metastasis. Patients presenting with metastatic disease at diagnosis were excluded from this analysis.

Secondary endpoints included progression-free survival (PFS) and duration of response (DoR). PFS was defined as the time from initiation of systemic therapy to radiographic or clinical progression or death from any cause. Progression was defined as new lesions, enlargement of existing lesions on imaging, or clinical deterioration attributable to tumor growth. Patients alive without documented progression at last follow-up were censored. DoR was defined as the interval from the first documentation of CR or PR to disease progression or last follow-up.

### Statistical analysis

Descriptive statistics were used to summarize patient demographics, clinical characteristics, histopathologic and molecular features, treatment regimens, and metastatic patterns. Continuous variables are presented as medians with interquartile ranges, and categorical variables as counts and percentages. Normality of continuous variables was assessed using the Shapiro–Wilk test. Given the non-normal distribution and limited sample size, continuous variables were compared using the Mann–Whitney *U* test. Categorical variables were compared using chi-square or Fisher’s exact tests, as appropriate.

Survival outcomes, including OS, time to distant metastasis, and PFS, were estimated using the Kaplan–Meier method, and survival curves were compared using the log-rank test.

For genomic analyses, an unsupervised gene-level oncoprint was generated. Genes and patients were hierarchically clustered based on a binary alteration matrix (any mutation or copy number alteration = 1, none = 0) using Jaccard distance and average linkage. This approach identifies co-altered genes and patient subgroups solely based on observed alteration patterns, without prior pathway assumptions. Pathway-level alteration frequencies in EMPD were compared with selected TCGA reference cohorts using Spearman’s rank correlation. All statistical analyses and data visualizations were performed in R version 4.5.2 (R Foundation for Statistical Computing, Vienna, Austria).

## Results

### Patients’ demographics

A total of 26 patients met the inclusion criteria and were included in the analysis. Of these, 18 patients (69.2%) had metastatic disease at initial presentation, while 8 (30.8%) developed distant recurrence following prior definitive local therapy. The median age at diagnosis was 68 years (IQR, 53-79), with 19/26 patients (73.1%) being male and a male-to-female ratio of 2.7:1. 92.3% of the patients were white (24/26), and 14/26 (53.9%) had a history of smoking (46.2% former, 7.7% current). An ECOG PS of 0-1 was recorded in 21/23 patients (91.3%). At the time of analysis, 65.4% of patients were deceased (17/26), with disease progression as the cause of death in 82.4% of cases (14/17). The median OS for the entire cohort was 20.0 months (95% CI, 0-40.4) with 5-year survival probability of 41.5%. Comparisons between patients with metastatic disease at presentation and those with distant recurrence are summarized in Table 1.

**Table 1.** Clinical and demographic comparisons between patients presenting with metastatic EMPD and those developing distant recurrence.

Variable	Total cohort *n* = 26 (%)	Metastatic at diagnosis, *n* = 18	Recurrent after definitive therapy, *n* = 8	*P* value
**Age (year): median, (IQR)**	68 (53-79)	73 (53-79)	65 (56-79)	**.05^a^**
**Gender**				
** Male**	19 (73.1%)	12 (66.7%)	7 (87.5%)	.4^b^
**Race**				
** White**	24 (92.3%)	16 (88.9%)	8 (100%)	.6^b^
**Smoking history**				
** Former**	12 (46.2%)	9 (50%)	3 (37.5%)	.8^b^
** Current**	2 (7.7%)	1 (5.6%)	1 (12.5%)	
**Performance status**				
** Good (0-1)**	21 (91.3%)	15 (88.2%)	6 (100%)	1^c^
**Primary tumor location**				
** Female Genital Region**	5 (19.2%)	4 (22.2%)	1 (12.5%)	.1^c^
** Male Genital Region**	7 (26.9%)	2 (11.1%)	5 (62.5%)	
** Groin Region**	8 (30.8%)	7 (38.9%)	1 (12.5%)	
** Perineal/Perianal Region**	5 (19.2%)	4 (22.2%)	1 (12.5%)	
** Other**	1 (3.8%)	1 (5.6%)		
**Stage at initial diagnosis**				
** I**	3 (11.5%)	0	3 (37.5%)	**.001^b^**
** II**	5 (19.2%)	0	5 (62.5%)	
** III**	10 (38.5%)	10 (55.6%)	0	
** IV**	8 (30.8%)	8 (44.4%)	0	
**Metastatic sites at diagnosis**				
** Liver**	1 (3.8%)	1 (5.6%)	–	1^c^
** LNs**	16 (61.5%)	16 (88.9%)	–	**.001** ^c^
** Bones**	5 (19.2%)	5 (27.8%)	–	.3^c^
** Muscle**	2 (7.7%)	2 (11.8%)	–	1^c^
**Presence of associated internal malignancy**				
** Yes**	1 (3.8%)	1 (5.6%)	0	1ᵈ
** No**	25 (96.2%)	17 (94.4%)	8 (100%)	
**Primary tumor size at diagnosis (cm): median, (IQR)**	7.8 (3-20)	7 (3-18)	9 (5-20)	.6^a^
**Multifocality**				
** Yes**	9 (24.6%)	4 (22.2%)	5 (62.5%)	.07^c^
** No**	17 (65.4%)	14 (77.8%)	3 (37.5%)	
**Associated visible features**				
** Erythema**	21 (80.8%)	14 (77.8%)	7 (87.5%)	1ᵈ
** Ulceration**	5 (19.2%)	4 (22.2%)	1 (12.5%)	
**Invasion at presentation**				
** In situ**	3 (11.5%)	0	3 (37.5%)	**.02^b^**
** Microinvasive (≤1 mm depth)**	1 (5.6%)	0	1 (12.5%)	
** Invasive (>1 mm depth)**	19 (71.4%)	15 (83.4%)	4 (50%)	
** Indeterminate/not reported**	3 (11.5%)	3 (16.7%)	0	
**Depth of invasion (mm): median (IQR)**	2 (0.8-18)	7.9 (8-15)	2 (1-18)	0.8^a^
**HER2 status**				
** Amplified**	10 (38.5%)	6 (33.3%)	4 (50%)	**.04^b^**
** Not amplified**	7 (26.9%)	6 (33.3%)	1 (12.5%)	
** Equivocal IHC**	2 (7.7%)	1 (5.6%)	1 (12.5%)	
** HER2 mutant**	2 (7.7%)	0	2 (25%)	
** NA**	5 (19.2%)	5 (27.8%)	0	
**Previous definitive therapy**				
** Yes**	15 (57.6%)	7 (38.8%)	8 (100%)	**.01^b^**
** No**	10 (38.5%)	10 (55.6%)	0	
** NA**	1 (3.8%)	1 (5.6%)	0	
**Median OS (months)**	20 (0.0-40.4)	20 (5.6-34.4)	15 (0.0-85.7)	.6^d^

Abbreviations: HER2, human epidermal growth factor receptor 2; IHC, immunohistochemistry.

Bold values indicate statistical significance (*P* < .05)

a Mann–Whitney.

b Chi-square.

c Fisher test.

d Log rank test.

### Clinical and pathological data

Primary tumor sites included the groin (8/26; 30.8%), male genital region (7/26; 26.9%), female genital region (5/26; 19.2%), and perineal or perianal region (5/26; 19.2%). The median primary tumor size was 7.8 cm (IQR, 3-20), and multifocal lesions were identified in (9/26; 34.6%) of patients. (21/26; 80.8%) of tumors exhibited erythema, while ulceration was noted in (5/26; 19.2%) of cases.

At initial diagnosis, (3/26; 11.5%) of patients had stage I disease, (5/26; 19.2%) stage II, (10/26; 38.5%) stage III, and (8/26; 30.8%) stage IV. Among those presenting with metastatic disease, the most common sites of involvement were lymph nodes (16/26; 61.5%), followed by bone (5/26; 19.2%), muscle (2/26; 7.7%), and liver (1/26; 3.8%). For patients initially diagnosed with localized disease who later developed distant recurrence, the median time to distant metastasis was 36 months (95% CI, 27-78), with distant lymph nodes (8/10; 80%), bone (7/10; 70%), liver (5/10; 50%), and lung (1/10; 10%) being the most frequently affected sites. Only one patient (3.8%) had an associated internal malignancy.

Invasive tumors were identified in all patients presenting with metastatic disease at initial diagnosis, while (4/8; 50%) of those who developed distant recurrence initially presented with invasive lesions, with a median invasion depth of 2 mm (IQR, 0.8-18). IHC analysis demonstrated universal positivity for cytokeratin 7 (26/26) and GATA3 (18/18; 100%), with gross cystic disease fluid protein 15 (GCDFP-15) expressed in 86.7% of evaluable cases (13/15) and mucicarmine positivity in 85.7% (6/7). Androgen receptor (AR) positivity was observed in 80% of tumors (8/10), whereas estrogen receptor (ER) expression was noted in 30.8% (4/13) and progesterone receptor (PR) was negative in 81.8% (9/11). HER2 was positive (3+) in 31.6% of cases (6/19), equivocal (2+) in 26.3% (5/19), and negative (0/1+) in 42.1% (8/19). Among cases with available FISH testing, HER2 amplification was identified in 42.9% (3/7). Within the equivocal IHC group, FISH demonstrated amplification in 20%, was negative in 40%, and was not performed in the remaining 40% (Table 2).

**Table 2.** HER2 status across different modalities (IHC, FISH, NGS).

HER2 IHC	FISH	NGS alteration
**NA**	NA	ND
**3+**	POS	Amplification
**3+**	NA	NA
**NA**	NA	ND
**2+**	NEG	RNA overexpression
**0**	NEG	ND
**3+**	NA	Amplification
**0**	NA	NA
**0**	NA	NA
**0**	NA	NA
**NA**	NA	NA
**NA**	NA	ND
**3+**	NA	ND
**1+**	NA	ND
**1+**	NEG	Amplification
**2+**	NA	NA
**3+**	NA	Amplification
**NA**	NA	ND
**2+**	NA	ND
**0**	NA	HER2 missense (GOF)
**2+**	POS	HER2 missense (GOF)
**3+**	NA	NA
**2+**	NEG	Amplification
**NA**	NA	HER2 missense (GOF)
**1+**	NA	NA
**NA**	POS	Amplification

Abbreviations: HER2, human epidermal growth factor receptor 2; IHC, immunohistochemistry; NGS, next-generation sequencing; LN, lymph node; ND, not detected; NA, not available; Neg, negative; GOF, gain-of-function.

**Figure 1.**
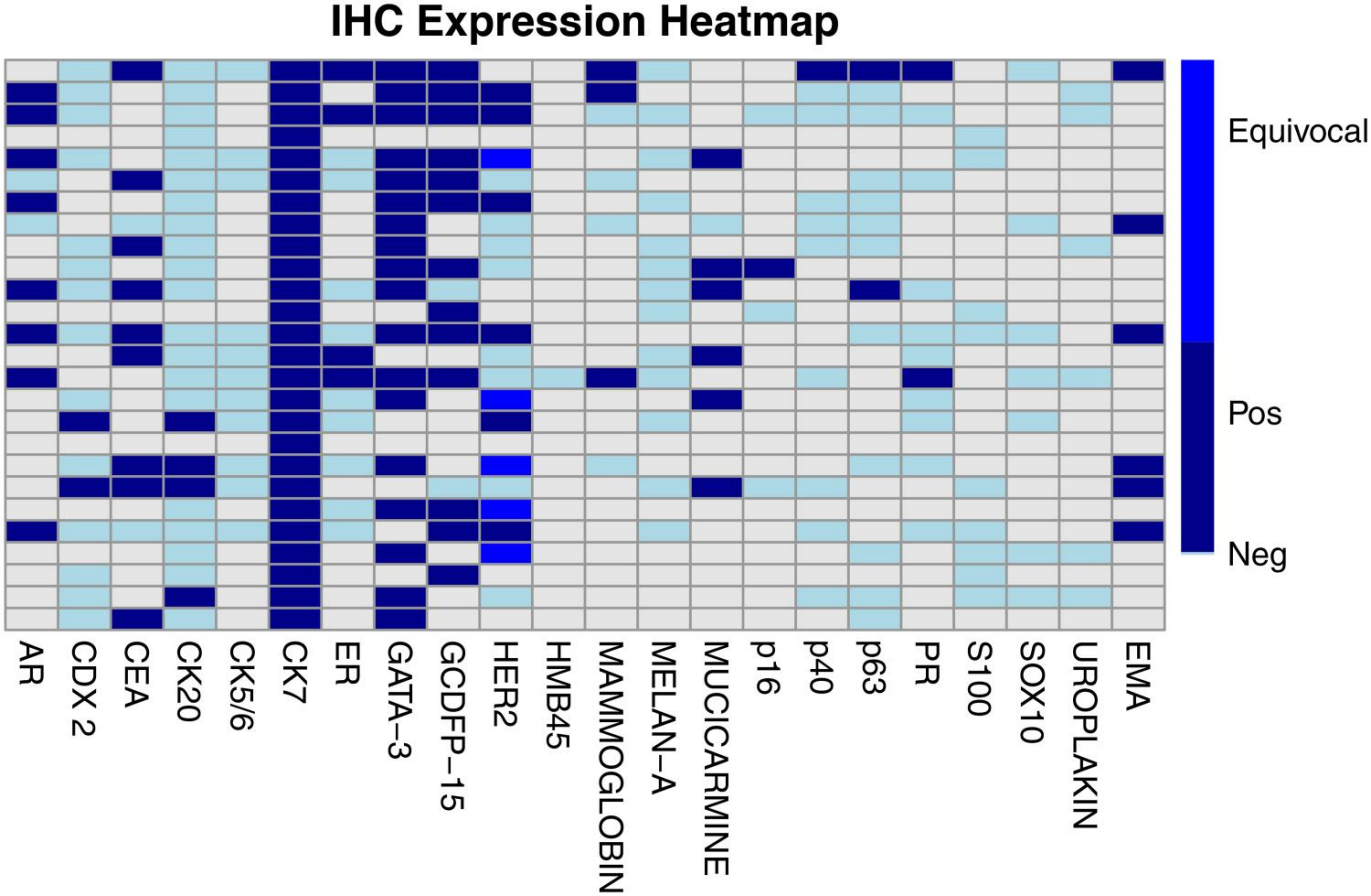
Immunohistochemical expression profile of metastatic and recurrent EMPD. Heatmap illustrating immunohistochemical marker expression across the cohort (*n* = 26). Each row represents an individual case and each column represents a marker. Staining status is categorized as positive expression (HER2 IHC 3+), equivocal expression (HER2 IHC 2+), negative expression (0–1+), or not tested/insufficient tissue.

CK5/6 was negative in 100% of cases (11/11), CK20 in 83.3% (20/24), CDX2 in 87.5% (14/16), p63 in 84.6% (11/13), and melanocytic markers (Melan-A, S100, and SOX10) were negative in all tested cases. Carcinoembryonic antigen (CEA) was positive in 81.8% of cases (9/11). Detailed immunohistochemical expression across the cohort is summarized in Figure 1, and the number of evaluable cases for each immunohistochemical marker is provided in Table S1.

### NGS data

NGS testing was available for 18 patients (69.2%), with tumor-based NGS performed in 16 of them (61.5%). TMB was high in 31.3% of cases, low in 56.3%, and not assessed in the remainder. Across all samples, a total of 183 genomic alterations were identified. The most frequently mutated genes were TP53 (68.8%), ERBB2 (HER2) (50%), CDKN2A (50%), PIK3CA (37.5%), and CDKN2B (31.3%). Additional recurrent alterations were detected in ERBB3, APC, and MTAP (each 25%), and in PML (18.8%) genes.

RNA expression data demonstrated recurrent overexpression of ERBB2 (HER2), ERBB3, MAPK1, EZH2, BRAF, RET, TOP2A, LAGE-1, and NY-ESO-1, and underexpression of CDKN2B.

**Figure 2.**
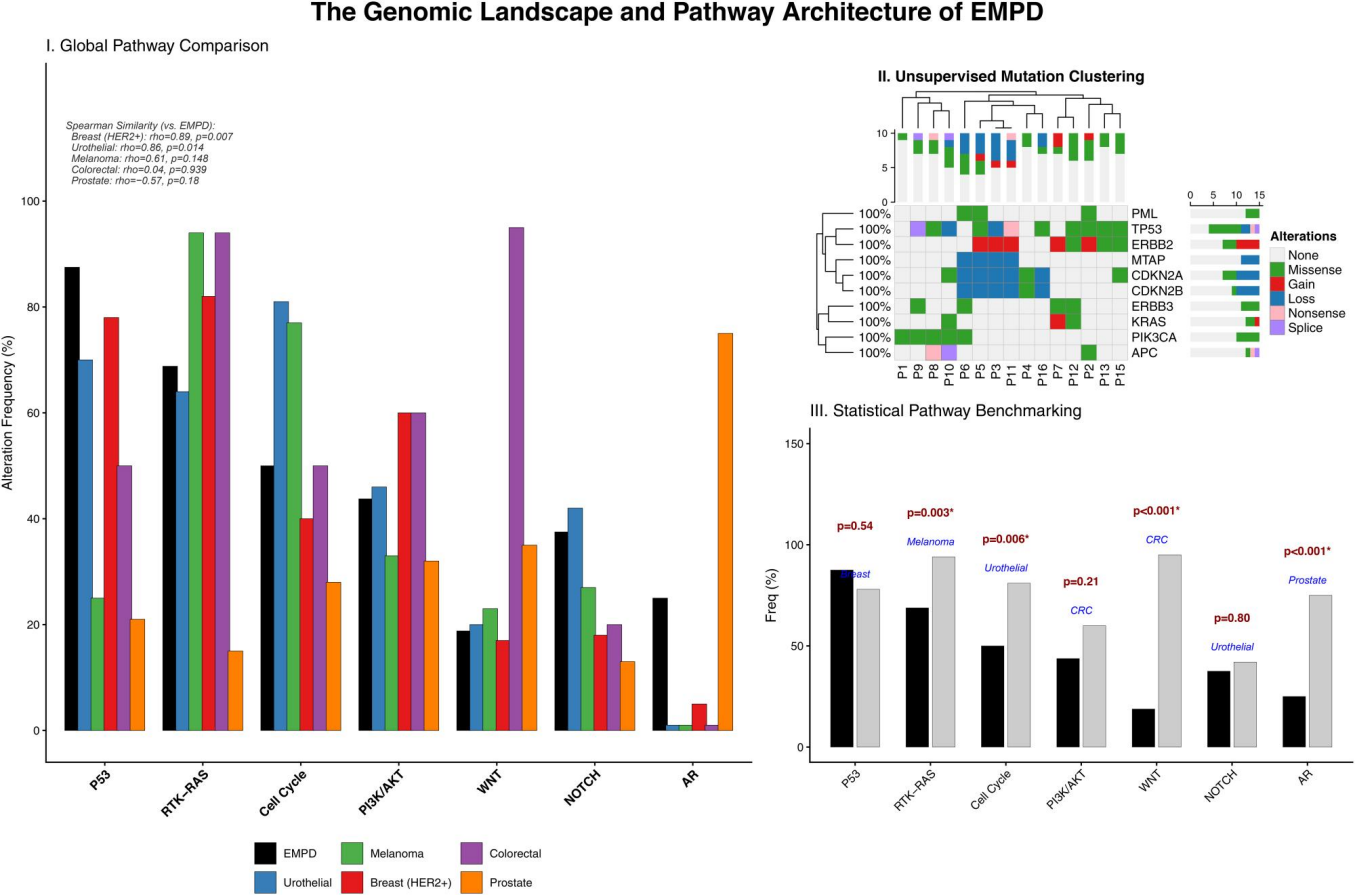
The genomic landscape of extramammary Paget’s disease and comparative pathway analysis. Panel I: Comparative Pathway Architecture. Bar plot illustrating the alteration frequencies (mutations and copy number variations) across 7 key oncogenic signaling pathways. EMPD (black, *n* = 16) is compared against 5 major malignancies (urothelial, melanoma, breast HER2+, colorectal, and prostate) derived from TCGA and reference cohorts. Spearman Rank Correlation (ρ) analysis in the upper-left quadrant quantifies the global pathway similarity between EMPD and the comparison cohorts, highlighting the high degree of genomic architectural overlap between EMPD and breast and urothelial. Panel II: Unsupervised Mutation Clustering. Clustered oncoPrint displaying the landscape of non-silent somatic mutations and copy number alterations in ten top-tier driver genes. Genes (rows) and Patients (columns) are organized via unsupervised hierarchical clustering using Jaccard distance (Ward.D2 method) based on binary alteration status (mutated vs wild-type). The dendrogram heights represent the genomic dissimilarity between samples. Panel III: Statistical Pathway Benchmarking. Direct comparison of EMPD alteration frequencies against specific TCGA benchmark malignancies. Each pathway in EMPD is statistically benchmarked against a specific tissue-of-origin proxy. *P*-values indicate the significance of the frequency difference between EMPD and the benchmark, as determined by Fisher’s exact test. Asterisks (*) denote statistical significance (*P* < .05).

Pathway-level analysis demonstrated frequent alterations in P53 signaling (87.5%), RTK–RAS pathways (68.8%), cell cycle regulation (50%), and PI3K signaling (43.8%), followed by NOTCH (37.5%) and WNT (18.8%) pathway alterations. Nuclear receptor/hormone signaling, including AR, was altered in 25% of cases. Spearman correlation analysis comparing EMPD to TCGA reference cohorts demonstrated strong similarity with HER2-positive breast carcinoma (ρ = 0.89, *P* = .007) and urothelial carcinoma (ρ = 0.86, *P* = .014), moderate similarity with melanoma (ρ = 0.61, *P* = .148), and little to no similarity with colorectal (ρ = 0.04, *P* = .939) or prostate carcinoma (ρ = −0.57, *P* = .18; Figure 2).

### Treatment data

Fifteen patients (57.6%) received definitive local therapy as initial treatment. Eight of these are those who initially had localized diseases but later developed distant recurrence, and 7 presented with stage III, locally advanced disease. Only patients who subsequently received systemic therapy are included in the analyses that follow. One patient did not receive any treatment at the time of the study and was excluded from the treatment analysis.

Ten patients received initial systemic therapy; regimens included taxane-based chemotherapy (3/10; 30%), 5-fluorouracil (5FU; 1/10; 10%), immune checkpoint inhibitors (ICI; 3/10; 30%), and HER2-directed therapy (3/10; 30%). Taxane-based chemotherapy yielded an ORR of 66.7% (2/3), with a median DoR1 of 21.5 months (95% CI, 7-36) and median PFS1 of 9 months (95% CI, 4-40). An ORR of 100% (2/3) was observed among patients who received HER2-directed therapy (one patient lost to follow-up), with a median DoR1 of 3.5 months (95% CI, 3-4) and median PFS1 of 6 months (95% CI, 4-7). In the ICI group, one patient achieved a PR, one had SD, and one experienced PD; the median PFS1 was 6 months (95% CI, 2-10).

The median number of subsequent systemic therapy lines was 1 (IQR, 1-7). Seventeen patients received second-line systemic therapy, including taxane-based regimens (6/17; 35.3%), HER2-directed therapy (7/17; 41.2%), ICI (1/17; 5.9%), 5FU-based regimens (1/17; 5.9%), and other regimens (2/17; 11.8%). Among those, Taxane-based regimens were associated with an ORR of 33.3% (2/6), with a median PFS2 of 3.5 months (95% CI, 2-53). HER2-directed therapy demonstrated an observed ORR of 71.4% (5/7), with a median DoR2 of 7 months (95% CI, 3-12) (some ongoing) and median PFS2 of 6 months (95% CI, 1-13).

**Figure 3.**
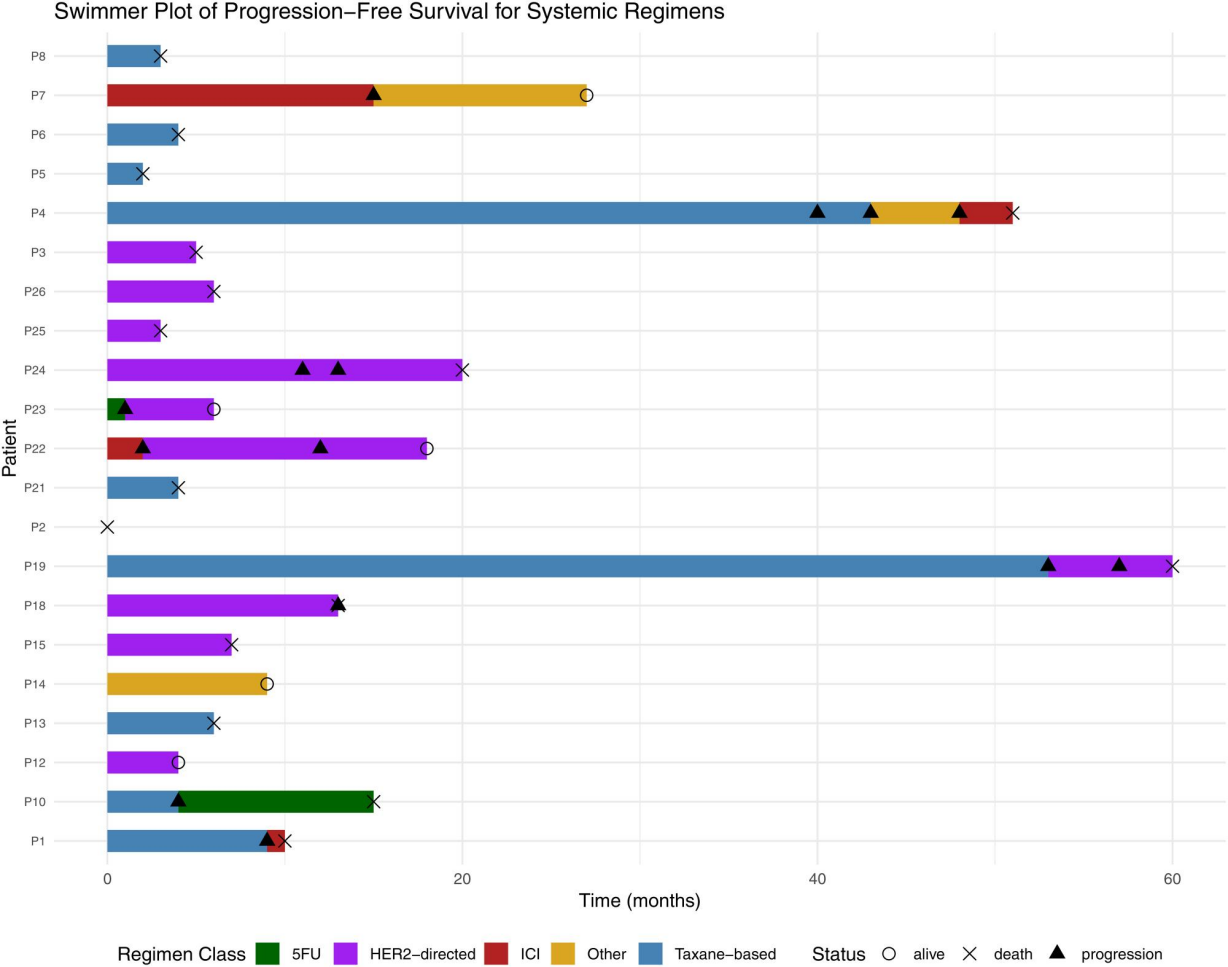
Swimmer plot of treatment duration and progression-free survival across systemic therapy lines. Each horizontal bar represents an individual patient’s treatment course for a given systemic regimen. Bar length indicates progression-free survival in months. Ongoing treatments at last follow-up are marked with a censoring symbol. Patient outcomes at last follow-up (alive, deceased, or progressed) are indicated by endpoint symbols.

Six patients received third-line therapy, which included 5FU-based regimens (2/6; 33.3%), HER2-directed agents (3/6; 50%), and gemcitabine (1/6; 16.7%). HER2-directed therapy was associated with an ORR of 33.3% (1/3) with a median PFS3 of 4 months (95% CI, 2-6). Full details of regimens, best responses, DoR, and PFS are provided in Table S2. PFS for the systemic therapy regimens is presented in Figure 3.

#### HER2-directed therapy

**Figure 4.**
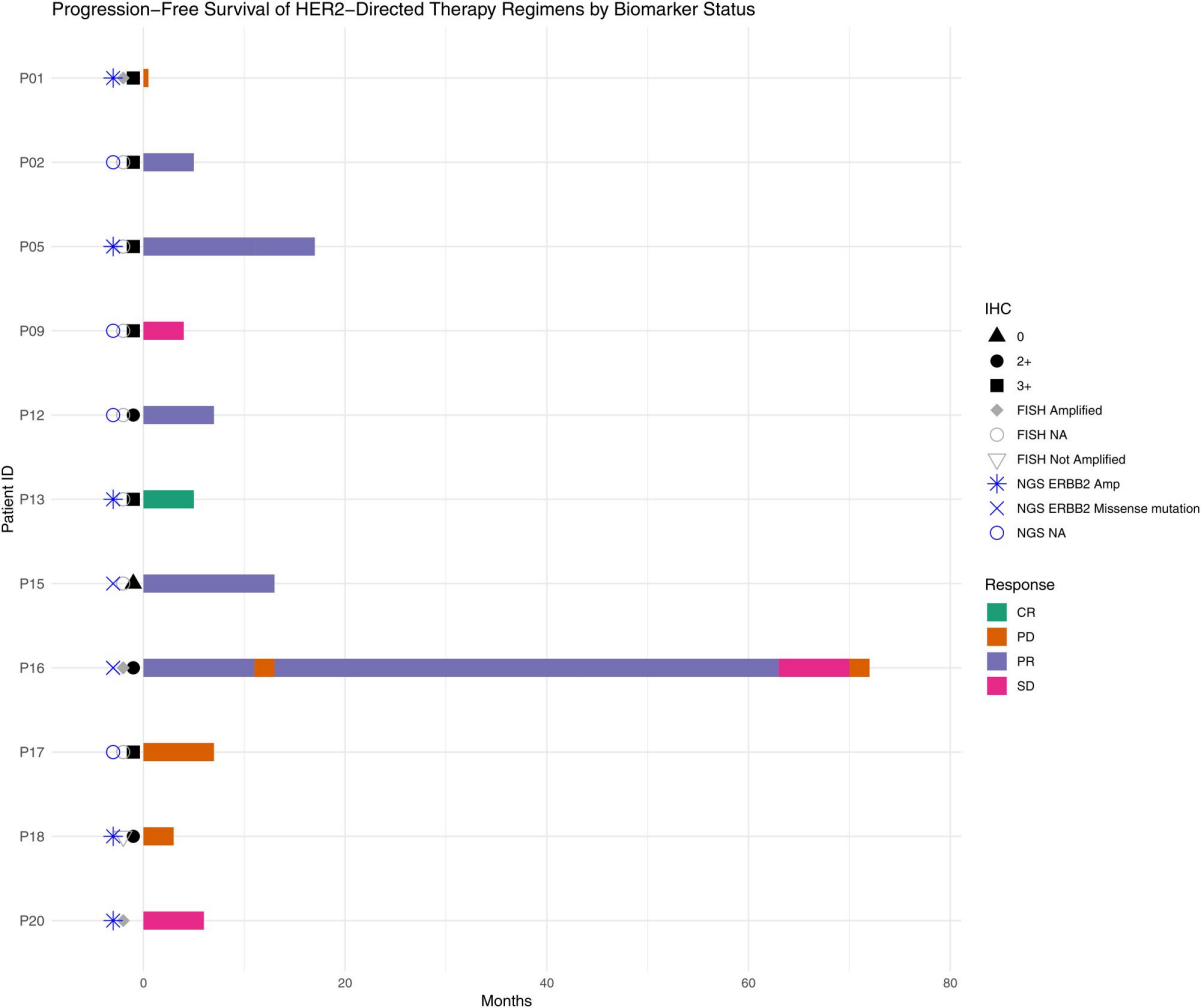
Swimmer plot of HER2-directed therapy outcomes stratified by integrated biomarker status. Each horizontal bar represents progression-free survival on HER2-targeted treatment. Bar length reflects treatment duration, with censoring indicated for ongoing therapy. Color indicates best objective response (complete response, partial response, stable disease, or progressive disease). Biomarker status (IHC, FISH, or NGS-based HER2 alteration) is annotated for each patient to correlate molecular features with clinical response.

Eleven patients received HER2-directed therapy during their disease course, including trastuzumab- or pertuzumab-based regimens, antibody–drug conjugates, tyrosine kinase inhibitors, and combinations with chemotherapy across multiple lines of treatment. Best responses included PR in 47.4%, CR in 5.3%, SD in 15.8%, and PD in 31.6%, with response and disease control durations varying by regimen and line. Patients who received HER2-directed therapy exhibited a numerically longer median OS (73.0 months [95% CI, 0-177.9] vs 19.0 months [95% CI, 0-41.4]; HR 0.5 [95% CI, 0.0-177.9], *P* = .3) and longer median second-line PFS (11.0 months [95% CI, 6.7-15.3] vs 4.0 months [95% CI, 1.1-6.9], *P* = .6) compared with those who did not receive HER2-directed therapy. However, these findings were not statistically significant and should be interpreted cautiously due to small sample size, selection bias, and lack of adjustment for confounders. Individual treatment regimens and outcomes are summarized in Table S3, and PFS stratified by HER2 biomarker status is shown in Figure 4.

## Discussion

In this study, metastatic EMPD comprised 11.2% of all EMPD cases identified at Mayo Clinic during the study period, a proportion consistent with rates reported in the SEER database.^30^ The male-to-female ratio in our cohort was 2.7:1. While SEER data suggest that metastatic EMPD overall—including cases with regional lymph node involvement—is more common in females (M: F ≈ 0.48), distant metastatic disease alone appears to occur slightly more frequently in men (2.6% vs 1.5%).^30^ A similar male predominance has been observed in metastatic EMPD in Asian cohorts, with reported ratios as high as 5:1,^31^ raising the possibility that EMPD arising in men may exhibit more aggressive biological behavior.^32^ The most common primary sites at diagnosis in our cohort were the groin, followed by the male genital and perineal/perianal regions. EMPD in these locations has been associated with deeper invasion, nodal metastases, and occasional underlying malignancies.^33–35^ Additionally, lesions in these areas are often subject to delayed recognition, which may contribute to more advanced disease at presentation and poorer clinical outcomes.^36^

Histopathologically, EMPD closely resembles mammary Paget disease, characterized by large, pale-staining Paget cells exhibiting glandular differentiation and often infiltrating adnexal structures.^3^ While most cases can be diagnosed morphologically, dermal invasion may obscure classic features and overlap with melanoma, Bowen’s disease, or sebaceous carcinoma. In such scenarios, mucin-specific stains such as PAS or Alcian blue can help highlight the mucinous cytoplasm typical of Paget cells.^37^ IHC further supports diagnosis and assists in distinguishing primary from secondary EMPD. Primary EMPD typically demonstrates positivity for CK7, GATA3, CEA, and GCDFP-15,^38^^,^^39^ and is usually negative for CK20 and CDX2,^40^ although site-specific immunophenotypic variations have been reported.^18^ This pattern was largely reflected in our cohort. Interestingly, focal CK20 and CDX2 expressions occurred in a small subset of cases, even though no clinical or pathological evidence of secondary EMPD was present, a finding that has been reported in primary EMPD and underscores the limited specificity of these markers when used in isolation.^18^

Hormone receptor expression in EMPD is generally more limited compared with breast cancer. In our cohort, ER positivity was observed in 30.8% of cases, higher than the ∼10% typically reported in EMPD,^41^ yet still markedly lower than in breast cancer. PR expression was largely absent (81.8%), consistent with prior reports.^41^ Conversely, AR positivity was observed in 80% of tumors, aligning with previous studies reporting high AR expression (54%-90%) in EMPD,^41^^,^^42^ particularly in invasive disease,^43^ supporting the hypothesis that androgen–AR signaling may contribute to tumor progression.^44^ Importantly, AR genomic alterations were present in only a minority of cases (25%), suggesting that AR pathway activation in EMPD may be predominantly driven by non-genomic mechanisms. Collectively, these findings suggest that AR may represent a more relevant therapeutic target than ER or PR in EMPD, supported by reports of responses to AR-directed therapy in metastatic disease.^45^^,^^46^

HER2 represents an important molecular alteration in EMPD, with reported positivity in approximately 10%-30% of cases.^47^^,^^48^^,^^49^ Standard HER2 assessment relies on IHC with reflex FISH testing for equivocal (2+) cases, while IHC 2+/FISH– tumors are conventionally classified as negative.^28^ Emerging evidence, however, suggests that some tumors with low or equivocal HER2 expression may still harbor activating ERBB2 alterations detectable by NGS.^50^^,^^51^ Consistent with this, several tumors in our cohort demonstrated ERBB2 amplification, activating mutations, or RNA overexpression despite low IHC expression or negative FISH results. These findings highlight HER2 heterogeneity in EMPD and support comprehensive molecular profiling in selected cases.

Our pathway-level analysis suggests that EMPD may share genomic features with systemic luminal malignancies rather than representing a purely localized cutaneous process. The observed Spearman similarity with HER2-positive breast and urothelial carcinomas supports a potential shared evolutionary trajectory and provides a rationale for basket-trial therapeutic strategies. We also identified a “triple-hit” deletion at the 9p21 locus, involving co-loss of CDKN2A, CDKN2B, and MTAP. In other solid tumors, this alteration is associated with aggressive behavior and poor prognosis^52^^,^^53^ but may also create therapeutic vulnerabilities. CDKN2A/B loss may result in cyclin D–CDK4/6 pathway activation, suggesting potential sensitivity to CDK4/6 inhibitors such as palbociclib or abemaciclib.^54^ MTAP loss may represent a collateral vulnerability with emerging therapeutic relevance to PRMT5 inhibitors currently under investigation in MTAP-deleted malignancies.^55^^,^^56^ However, these implications are extrapolated from other cancers and remain hypothesis-generating pending functional and clinical validation in EMPD.

Treatment patterns in our cohort were highly heterogeneous, reflecting the lack of standardized systemic therapy for this rare malignancy. Platinum- and taxane-based chemotherapies were commonly used in first- and second-line settings, yielding occasional durable responses but overall modest efficacy. In comparison, HER2-directed therapies were associated with radiographic responses and prolonged observed disease control in several patients, including in later lines of therapy. Notably, progression on one HER2-targeted agent did not preclude benefit from subsequent HER2-directed therapies, with one patient achieving cumulative disease control exceeding 70 months through sequential treatment. Trastuzumab deruxtecan (T-DXd), an antibody–drug conjugate, is FDA-approved for previously treated unresectable or metastatic solid tumors regardless of histology in selected contexts, based in part on the DESTINY-PanTumor02 phase II basket trial, which included patients with EMPD and demonstrated activity in HER2-expressing rare tumors.^57^ Beyond T-DXd, small-molecule inhibitors and Fc-engineered antibodies have also demonstrated efficacy in HER2-driven malignancies, highlighting additional therapeutic options.^58–60^ Despite the limitations of this retrospective design and small cohort size, patients receiving HER2-directed therapy demonstrated longer observed OS and PFS compared with those who did not receive HER2-directed therapy. However, these differences did not reach statistical significance and should be interpreted cautiously. Larger multi-institutional studies of HER2-directed therapy in metastatic EMPD are needed.

Importantly, responses to HER2-directed therapies were observed not only in IHC 3+/FISH-amplified tumors but also in tumors with low or equivocal HER2 expression, including non-amplified tumors harboring activating ERBB2 mutations. These findings align with breast cancer trials demonstrating the biological relevance and targetability of such mutations.^61–63^ By contrast, ICIs showed limited activity, with predominantly stable disease or progression, reinforcing prior observations that EMPD may be intrinsically less responsive to checkpoint blockade, even in biomarker-positive tumors.^64^^,^^65^

This retrospective study is limited by its small cohort size, treatment heterogeneity, and extended study period, which introduced variability in diagnostic practices, imaging, molecular testing, and systemic therapies. Response assessment was based on retrospective radiology review rather than RECIST criteria, potentially affecting ORR estimation. Multivariable adjustment was not feasible because of the limited sample size and number of events. In addition, immunohistochemical markers were not uniformly assessed across cases, introducing variable denominators and potential selection bias. As a tertiary referral center cohort enriched for metastatic and recurrent disease, referral and treatment-selection bias may also limit generalizability. Accordingly, findings should be considered descriptive and hypothesis-generating.

Despite these limitations, this study represents one of the most detailed cohorts of metastatic and recurrent EMPD, integrating clinicopathologic, genomic, and real-world treatment data. Our findings suggest clinical activity of HER2-directed therapies, including tumors with equivocal IHC/FISH results, supporting the role of comprehensive molecular profiling in therapeutic selection. Frequent AR expression and 9p21 (CDKN2A/B/MTAP) co-deletion identify additional potential therapeutic vulnerabilities warranting further investigation. Genomic similarities between EMPD and HER2-positive breast and urothelial carcinomas further support biomarker-driven treatment strategies. Importantly, this study focuses specifically on metastatic and distantly recurrent EMPD and should not be extrapolated to the broader EMPD population, which is typically diagnosed at an in situ or localized stage with favorable outcomes following local therapy.

## Supplementary Material

oyag220_Supplementary_Data

## Data Availability

The data that support the findings of this study are available from the corresponding author upon reasonable request. The data are not publicly available due to privacy or ethical restrictions, as they contain information that could compromise the privacy of research participants in accordance with HIPAA regulations and Mayo Clinic Institutional Review Board policies.
