# Supplementary material for: Clinicopathologic, molecular, and treatment features of metastatic and distantly recurrent extramammary Paget disease: Mayo clinic experience
Source: Oncologist. 2026 Jun 2;31(7):oyag220. doi: 10.1093/oncolo/oyag220 (PMC13283476; doi:10.1093/oncolo/oyag220)
Supplement: oyag220_Supplementary_Data [file oyag220_supplementary_data.zip › Supplementary_Tables.docx]

| **Marker** | **Tested (N)** | **Positive n (%)** | **Negative n (%)** | **Equivocal / Notes** |
| --- | --- | --- | --- | --- |
| CK7 | 26 | 26 (100) | 0 (0) | — |
| GATA3 | 18 | 18 (100) | 0 (0) | — |
| GCDFP-15 | 15 | 13 (86.7) | 2 (13.3) | — |
| Mucicarmine | 7 | 6 (85.7) | 1 (14.3) | — |
| AR | 10 | 8 (80.0) | 2 (20.0) | — |
| ER | 13 | 4 (30.8) | 9 (69.2) | — |
| PR | 11 | 2 (18.2) | 9 (81.8) | — |
| HER2 (IHC) | 19 | 6 (31.6, 3+) | 8 (42.1, 0–1+) | 5 (26.3, 2+) |
| HER2 (FISH) | 7 | 3 (42.9 amplified) | 4 (57.1 non-amplified) | Performed in selected cases |
| CK5/6 | 11 | 0 (0) | 11 (100) | — |
| CK20 | 24 | 4 (16.7) | 20 (83.3) | — |
| CDX2 | 16 | 2 (12.5) | 14 (87.5) | — |
| p63 | 13 | 2 (15.4) | 11 (84.6) | — |
| Melan-A | 13 | 0 | 13 (100) | — |
| S100 | 9 | 0 | 9 (100) | — |
| SOX10 | 7 | 0 | 7 (100) | — |
| CEA | 11 | 9 (81.8) | 2 (18.2) | — |
| HMB45 | 1 | 0 | 1 (100) | — |
| Mammoglobin | 7 | 3 (42.9) | 4 (57.1) | — |
| P16 | 4 | 1 (25) | 3 (75) | — |
| P40 | 10 | 1 (10) | 0 (90) | — |
| Uroplakin | 6 | 0 | 6 (100) | — |
| EMA | 6 | 6 (100) | 0 | — |

**Supplementary Table 1: Immunohistochemical and HER2 Testing Results in Metastatic and Distantly Recurrent Extramammary Paget Disease**

**Supplementary Table2*. Full details of treatment lines among our cohort**

| **Patient** | **First line** | | | | **Second line** | | | | **Third line** | | | | **Fourth line** | | | |
| --- | --- | --- | --- | --- | --- | --- | --- | --- | --- | --- | --- | --- | --- | --- | --- | --- |
|  | **Agent** | **B.R** | **DOR1** | **PFS1/RFS** | **Agent** | **Best response** | **DOR2** | **PFS2** | **Agent** | **B.R** | **DOR3** | **PFS3** | **Agent** | **B.R** | **DOR4** | **PFS4** |
| 1 | carboplatin-paclitaxel | PR | 7.0 | 9.0 | pembrolizumab | PD |  | 1.0 |  |  |  |  |  |  |  |  |
| 2 | paclitaxel-trastuzumab | L-F_U |  | NA |  |  |  |  |  |  |  |  |  |  |  |  |
| 3 | TDX | PR | 3.0 | 5.0 |  |  |  |  |  |  |  |  |  |  |  |  |
| 4 | carboplatin-paclitaxel | CR | 36.0 | 40.0 | carboplatin-paclitaxel | PR | 1.0 | 3.0 | gemcitabine | PR | 1.0 | 5.0 | pembrolizumab | PD |  | 3.0 |
| 5 | Surgery | CR |  | 86.0 | docetaxel | PD |  | 2.0 |  |  |  |  |  |  |  |  |
| 6 | Surgery | PD |  | .0 | carboplatin-paclitaxel | SD |  | 4.0 |  |  |  |  |  |  |  |  |
| 7 | pembrolizumab | SD |  | 15.0 | thalidomide | PR | 7.00/ongoing | 12.0 |  |  |  |  |  |  |  |  |
| 8 | Surgery RT | CR |  | 27.0 | carboplatin-docetaxel | SD |  | 3.0 |  |  |  |  |  |  |  |  |
| 9 | Surgery | CR |  | 51.0 |  |  |  |  |  |  |  |  |  |  |  |  |
| 10 | nab paclitaxel | SD |  | 4.0 | 5FU | SD |  | 11.0 |  |  |  |  |  |  |  |  |
| 11 | RT | L-F-U |  |  |  |  |  |  |  |  |  |  |  |  |  |  |
| 12 | Surgery | PD |  | .0 | TDX | CR | ongoing | 4.0 |  |  |  |  |  |  |  |  |
| 13 | carboplatin-paclitaxel-pembrolizumab | PR | 3.0 | 6.0 |  |  |  |  |  |  |  |  |  |  |  |  |
| 14 | Surgery Cisplatin with RT | CR | 8.0 | 11.0 | ribociclib | CR | 8.00/ongoing | 9.0 |  |  |  |  |  |  |  |  |
| 15 | TDX | PR | 4.0 | 7.0 |  |  |  |  |  |  |  |  |  |  |  |  |
| 16 | NA |  |  |  |  |  |  |  |  |  |  |  |  |  |  |  |
| 17 | Surgery | CR |  | 262.0 |  |  |  |  |  |  |  |  |  |  |  |  |
| 18 | Surgery | CR |  | 78.0 | trastuzumab-pertuzumab | PR | 12.00 | 13.0 | CAPTEM | PD |  | 0 |  |  |  |  |
| 19 | Surgery | CR |  | 92.0 | carboplatin-docetaxel | CR | 50.00 | 53.0 | capecitabine-lapatinib | PD |  | 4.0 | ado-trastuzumab emtansine | PD |  | 3.0 |
| 20 | Surgery | PD |  | 12.0 |  |  |  |  |  |  |  |  |  |  |  |  |
| 21 | Surgery | CR |  | 32.0 | carboplatin-docetaxel | SD |  | 4.0 |  |  |  |  |  |  |  |  |
| 22 | pembrolizumab | PD |  | 2.0 | paclitaxel-trastuzumab | PR | 10.00 | 11.0 | TDX | PR | 4.0/ongoing | 6.0 |  |  |  |  |
| 23 | FOLFIRINOX | PD |  | 1.0 | docetaxel-trastuzumab-pertuzumab | CR | 3.00/ongoing | 5.0 | Capecitabine | SD |  | 2.0 |  |  |  |  |
| 24 | Surgery | CR |  | 49.0 | paclitaxel-trastuzumab | PR | 7.00 | 11.0 | neratinib | PD |  | 2.0 | ado-trastuzumab emtansine | PR | 6.0 | 7.0 |
| 25 | Surgery | CR |  | 39.0 | docetaxel-trastuzumab | PD |  | 3.0 |  |  |  |  |  |  |  |  |
| 26 | carboplatin-paclitaxel with RT | CR | 21.0 | 24.0 | docetaxel-trastuzumab-pertuzumab | SD |  | 6.0 |  |  |  |  |  |  |  |  |

*: B.R: Best Response, DOR: Duration of Response, PFS: Progression-Free Survival, RFS: Recurrence-Free Survival, PD: Progressive Disease, PR: Partial Response, CR: Complete Response, SD: Stable Disease, TDX: Trastuzumab Deruxtecan, RT: Radiation Therapy, CRT: Chemoradiation Therapy, NA: Not Available

**Supplementary Table3*.** **Individual Her2-directed regimens, responses, and outcomes by biomarker**

| **Patient ID** | **HER2 IHC** | **FISH** | **NGS** | **HER2 Therapies (with line)** | **Best response (per line)** | **DOR (mo) (per line)** | **PFS (mo)** |
| --- | --- | --- | --- | --- | --- | --- | --- |
| P01 | 3+ | Amplified | ERBB2 Amplification | I: paclitaxel-trastuzumab | DP | - | 0.5 |
| P02 | 3+ | NA | NA | I: TDX | PR | 3.0 | 5.0 |
| P05 | 3+ | NA | ERBB2 Amplification | II: paclitaxel-trastuzumab  III: TDX | PR  PR | 10.0  4.0 | 11.0  6.0 |
| P09 | 3+ | NA | NA | II: TDX | SD | - | 2.0 |
| P12 | 2+ | NA | NA | I: TDX | PR | 4.0 | 7.0 |
| P13 | 3+ | NA | ERBB2 Amplification | II: docetaxel-trastuzumab-pertuzumab | CR | 3.0 | 5.0 |
| P15 | 0 | NA | ERBB2 Missense mutation | II: trastuzumab-pertuzumab | PR | 12.0 | 13.0 |
| P16 | 2+ | Amplified | ERBB2 Missense mutation | II: paclitaxel-trastuzumab | PR | 7.0 | 11.0 |
|  |  |  |  | III: neratinib | DP | - | 2.0 |
|  |  |  |  | IV: ado-trastuzumab emtansine | PD | 5.5 | 7.0 |
|  |  |  |  | V: capecitabine-trastuzumab-tucatinib | PR | 19.0 | 22.0 |
|  |  |  |  | VI: TDX | PR | 16.0 | 19.0 |
|  |  |  |  | VII: margetuximab-navelbine | SD | - | 7.0 |
|  |  |  |  | VIII: gemcitabine-trastuzumab-lapatinib | DP | - | 3.0 |
| P17 | 3+ | NA | NA | III: capecitabine-lapatinib  IV: ado-trastuzumab emtansine | DP  DP | -  - | 4.0  3.0 |
| P18 | 2+ | Not Amplified | ERBB2 Amplification | II: docetaxel-trastuzumab | DP | - | 3.0 |
| P20 | NA | Amplified | ERBB2 Amplification | II: docetaxel-trastuzumab-pertuzumab | SD | - | 6.0 |

*: IHC: immunohistochemistry, NGS: next generation sequencing, DOR: Duration of Response, PFS: Progression-Free Survival, PD: Progressive Disease, PR: Partial Response, CR: Complete Response, SD: Stable Disease, TDX: Trastuzumab Deruxtecan, mo: months, NA: Not Available
