# Supplementary figures and images for: Clinicopathologic, molecular, and treatment features of metastatic and distantly recurrent extramammary Paget disease: Mayo clinic experience
Source: Oncologist. 2026 Jun 2;31(7):oyag220. doi: 10.1093/oncolo/oyag220 (PMC13283476; doi:10.1093/oncolo/oyag220)

**Supplementary Figure1.** **Flow diagram of case inclusion in our cohort**


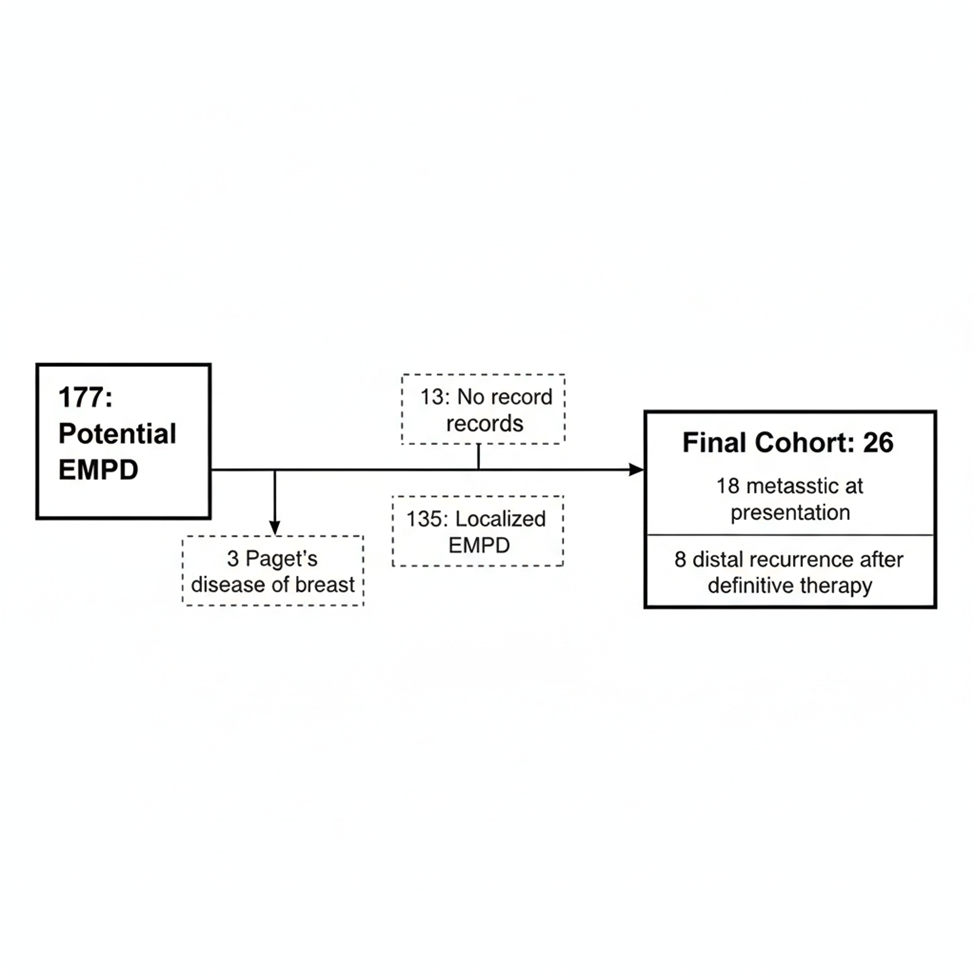

Supplement: oyag220_Supplementary_Data [file oyag220_supplementary_data.zip › Supplementary_Figure.docx]
